# Failed pneumoperitoneum for laparoscopic surgery following autologous Deep Inferior Epigastric Perforator (DIEP) flap breast reconstruction: a case report

**DOI:** 10.1186/s12893-016-0143-4

**Published:** 2016-04-27

**Authors:** Daniel M Balkin, Quan-Yang Duh, Gabriel M Kind, David S Chang, Mary H McGrath

**Affiliations:** Department of Surgery, Division of Plastic and Reconstructive Surgery, University of California San Francisco, San Francisco, CA USA; Department of Surgery, Section of Endocrine Surgery, University of California San Francisco, San Francisco, CA USA; Department of Plastic Surgery, California-Pacific Medical Center, San Francisco, CA USA

**Keywords:** Abdominal insufflation, Breast reconstruction, Endocrine surgery, Microsurgery, Minimally invasive surgery

## Abstract

**Background:**

Laparoscopic abdominal surgery may prove difficult in patients who have undergone previous abdominal procedures. No reports in the medical literature have presented an aborted laparoscopic procedure for failed pneumoperitoneum following autologous flap-based breast reconstruction.

**Case presentation:**

A 55-year-old woman presented with recurrent invasive lobular carcinoma of the right breast as well as a history of ductal carcinoma in situ of the left breast. The patient desired to proceed with bilateral skin- and nipple-sparing mastectomies with right axillary lymph node biopsy, followed by immediate bilateral autologous deep inferior epigastric perforator (DIEP) flap-based breast reconstruction. Preoperatively, a computerized tomography angiogram was obtained for reconstructive preparation, which revealed a left adrenal mass. Ensuing work-up diagnosed a pheochromocytoma. Given the concern for breast cancer progression, the patient elected to proceed first with breast cancer surgery and reconstruction prior to addressing the adrenal tumor. Subsequently, 3 months later the patient was brought to the operating room for a laparoscopic left adrenalectomy for the pheochromocytoma. With complete pharmacologic abdominal relaxation, the abdomen proved too tight to accommodate sufficient pneumoperitoneum and the laparoscopy was aborted. The patient was evaluated in the outpatient setting for assessment of abdominal wall compliance at regular intervals. Five months later, the patient was taken back to the operating room where pneumoperitoneum was established without difficulty and the laparoscopic left adrenalectomy was performed without complications.

**Conclusion:**

Pneumoperitoneum for laparoscopic surgery subsequent to autologous DIEP flap-based breast reconstruction may prove difficult as a result of loss of abdominal wall compliance. Prior to performing laparoscopy in such patients, surgeons should consider the details of the patient’s previous reconstructive procedure and assess potential risk factors for difficulty with insufflation. Lastly, careful abdominal examination should be performed to indicate whether laparoscopy for elective procedures should be delayed until abdominal wall compliance normalizes.

## Background

Laparoscopic abdominal surgery may prove difficult in patients who have undergone previous abdominal procedures. Routine anatomic landmarks may be distorted rendering safe trocar placement challenging. Intra- and retro-peritoneal scaring may increase technical difficulty. In addition, loss of abdominal wall compliance caused by abdominal wall fibrosis, removal of skin and soft tissue, fascial plication or the use of synthetic material may result in inadequate pneumoperitoneum [1–6].

Minimally invasive laparoscopic surgery is one of the most commonly performed procedures in general surgery with over 2 million cases performed annually in the United States [7]. The prevalence of breast reconstruction surgery following mastectomy for breast cancer has also increased [8–11]. In 2013, the American Society of Plastic Surgeons reported that 95,589 breast reconstructive procedures were performed [12]. Various breast reconstructive techniques exist, including several that involve autologous tissue transfer from the anterior abdominal wall.

The deep inferior epigastric perforator (DIEP) flap is a frequent autologous-based breast reconstructive option [12]. In this procedure, the deep inferior epigastric perforating artery and vein, along with the skin and soft tissues supplied by these vessels, is transferred to the chest wall with microvascular anastomoses to reconstruct the breast mound [13, 14].

We are presenting the first reported case of failed pneumoperitoneum for laparoscopy in a patient who had undergone previous DIEP flap-based breast reconstruction. Recommendations for recognizing and addressing loss of abdominal wall distensibility in patients who have had breast reconstruction with abdominal wall tissue are discussed.

## Case presentation

A 55-year-old woman presented with recurrent invasive lobular carcinoma of the right breast following previous lumpectomy and partial irradiation for invasive lobular carcinoma. Ductal carcinoma in situ of the opposite left breast also had been treated in the past with lumpectomy. Given the patient history and the recurrence of disease in the right breast, bilateral skin- and nipple-sparing mastectomies with right axillary sentinel lymph node biopsy were planned. These would be followed by immediate bilateral autologous DIEP flap-based breast reconstructions.

Preoperatively, a computerized tomography angiogram was obtained to evaluate the perforator vascular anatomy of the anterior abdominal wall. This study showed a 2.5–3.0-cm left adrenal mass. Subsequent work-up diagnosed a pheochromocytoma. The patient was offered a laparoscopic adrenalectomy after alpha-blockade in addition to a genetic evaluation for hereditary causes of pheochromocytoma. She was advised to undergo an adrenalectomy first before moving forward with oncologic and reconstructive breast surgery. However, given her concern for breast cancer progression, the patient preferred to proceed first with the breast cancer surgery and reconstruction to be followed with later surgery to address the adrenal tumor.

**Fig. 1:**
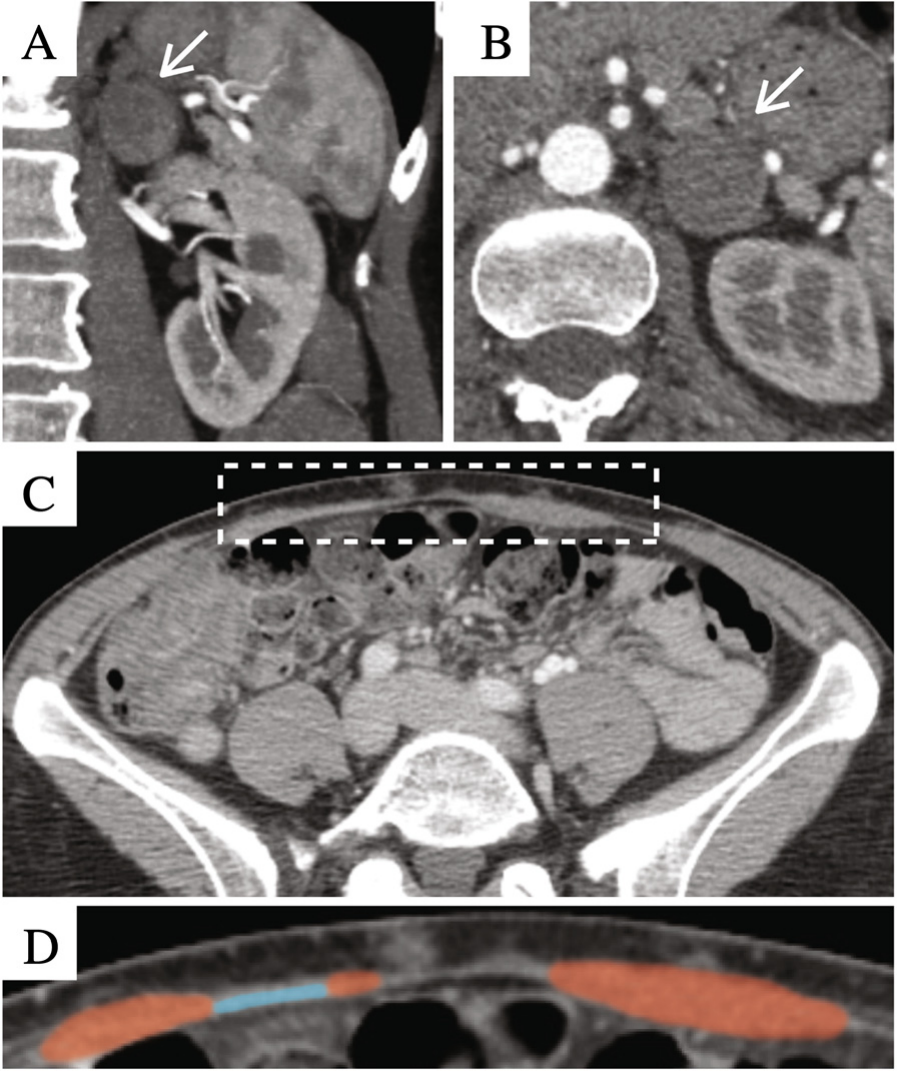
Computed Tomographic Imaging Prior to Laparoscopy Following Breast Surgery. **a** Coronal and **b** axial images demonstration left-sided pheochromocytoma. **c** Low-magnification axial image of the abdomen (white box highlights anterior abdominal wall). **d** High-magnification axial image of the anterior abdominal wall (red and blue indicate rectus abdominis muscle and mesh, respectively)

Under consultation with the patient’s Endocrinologist, alpha-blockade (phenoxybenzamine) was initiated 2 weeks before surgery to prevent pheochromocytoma crisis and beta blockage (propanolol) was started 1 week later for heart rate control. Subsequently, bilateral skin- and nipple-sparing mastectomies were initiated with simultaneous abdominal tissue harvest for the reconstruction. Figure 1 a Doppler probe was used to identify the dominant perforators of the abdominal wall on each side of the midline. As the skin and soft tissue flap was developed, dissection on the right side in the suprafascial plane showed the perforating vessels to be small, measuring less than 1.0-mm in diameter. To avoid injury to these vessels, the customary transmuscular dissection of the vessels was changed to include in the flap a 4 × 3-cm portion of the right rectus abdominis muscle and 4 × 2-cm portion of rectus fascia to surround and protect the vessels. On the left side, the perforating vessels to the flap also were small, but only a small cuff of rectus abdominis muscle and fascia was included in the flap. On both sides, only the deep inferior epigastric vessels were used for blood supply.

**Fig. 2:**
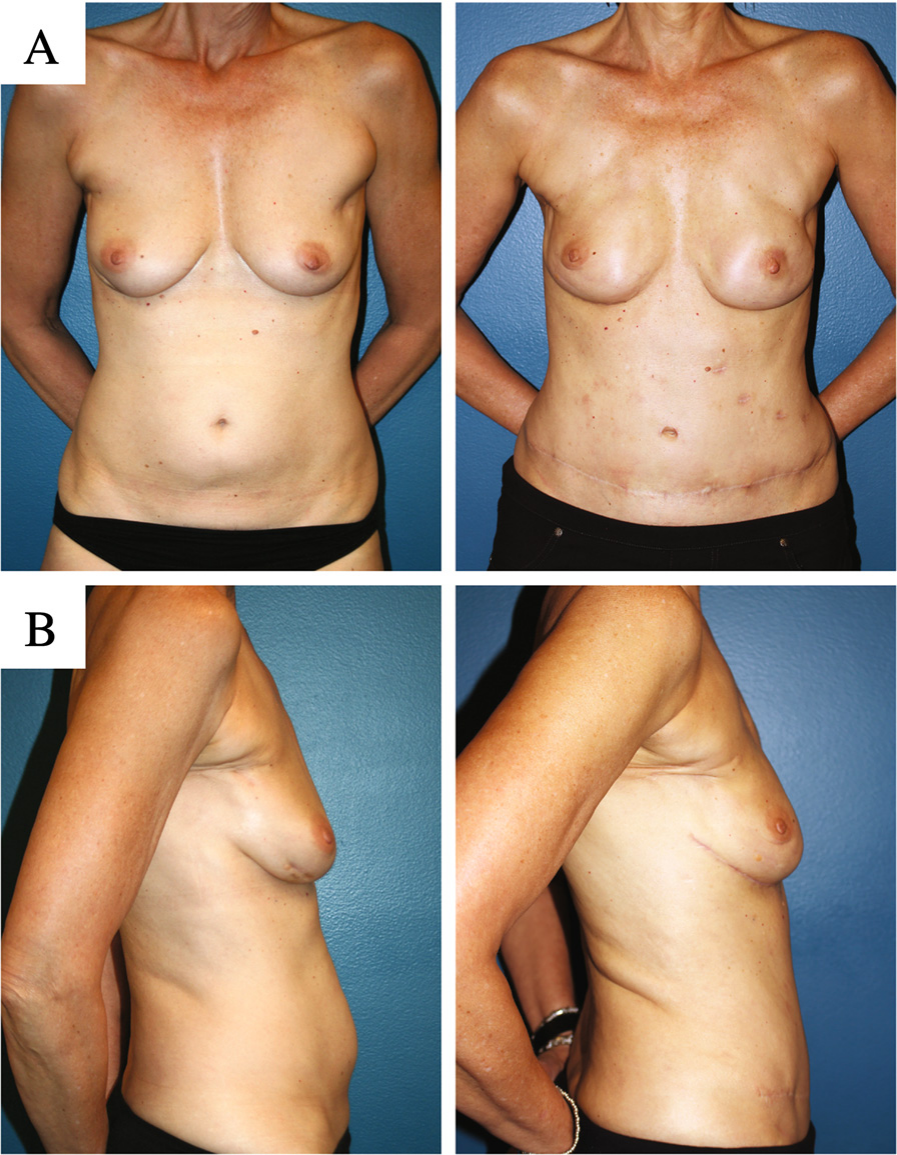
Patient Photographs Pre and Post Breast Cancer Surgery and Reconstruction. **a** Frontal and **b** lateral views (*left*, pre-operative; *right*, post-operative). Note degree of abdominal wall tightening post-operatively

Following completion of the bilateral mastectomies, the internal mammary arteries and veins were isolated by removing a short segment of the third costal cartilage. The deep inferior epigastric vessels of the abdominal flaps were divided and microvascular anastomoses were performed between the deep inferior epigastric artery and vein and the internal mammary artery and vein on each side. With strong flow through the anastomoses, the flaps were deepithelialized (with the exception of a small skin paddle along the inframammary fold for postoperative monitoring), and inset in the mastectomy defects. The abdominal wall donor sites were then closed. Because the 4 × 3 cm segment of rectus abdominis muscle and fascia had been included on the right side to protect the vessels, the fascial closure was reinforced with a 4 × 6-cm piece of SeriScaffold® mesh (knitted, multi-filament, bioengineered silk) [15]. The mesh was placed as an underlay deep to the rectus fascia secured with 1 PDS mattress sutures, and the fascial edges were then approximated over the mesh with 0 Ethibond figure-of-eight sutures. On the left side, the rectus abdominis fascial closure was completed with 0 Ethibond and 1 PDS sutures. In order to prevent bulging in the upper abdomen where tissue had not been removed, the fascia was tightened by placating with an 0 PDS running suture. The remaining abdominal skin was advanced and closed in layers with exteriorization of the umbilicus through the skin in the midline (Fig. 2).

**Fig. 3:**
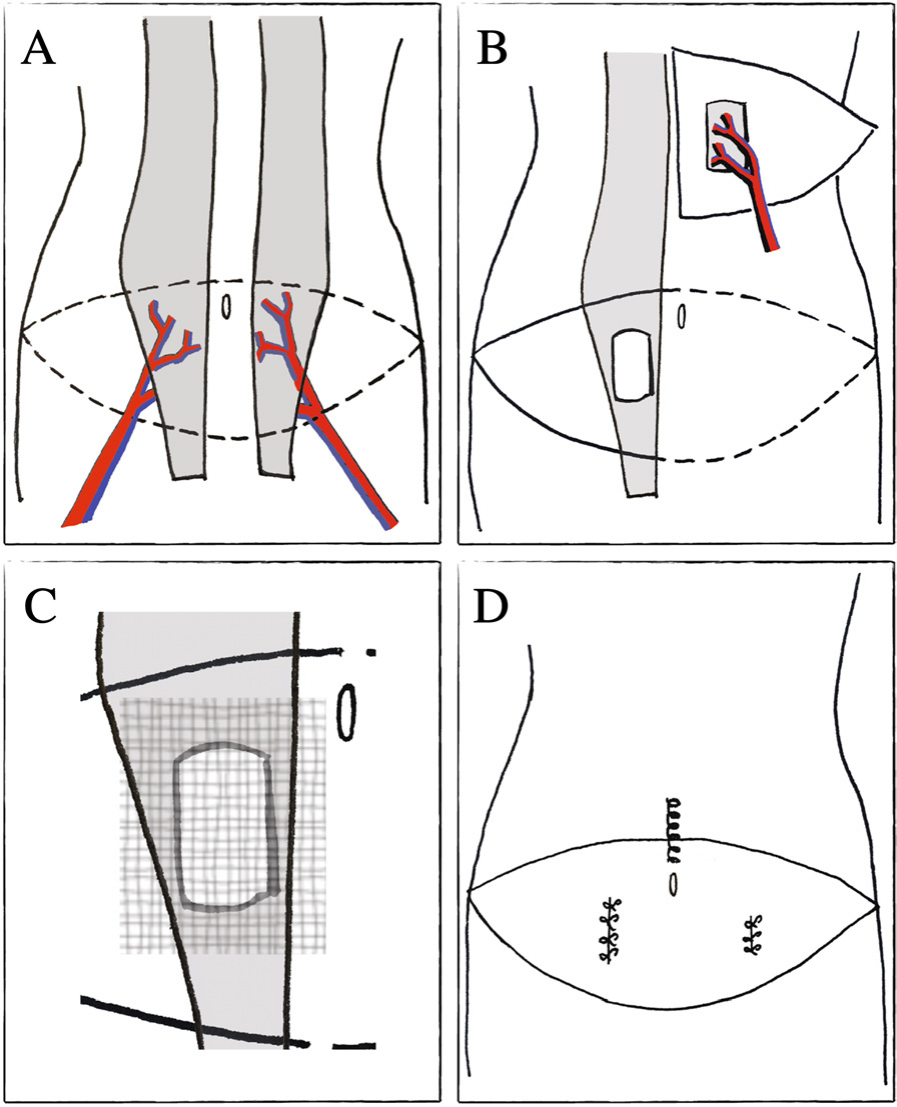
Illustrations of Various Operative Stages of Patient’s Breast Reconstruction. **a** Abdominal wall depicting bilateral rectus abdominis muscles (*grey*) with associated deep inferior epigastric arteries (*red*) and veins (*blue*). Dashed line indicates skin and soft tissue flaps harvested for breast reconstruction; **b** Right-sided DIEP flap used to recreate the left breast mound superimposed over left chest wall. Flap includes a portion of the right rectus abdominis muscle (*grey*) and rectus fascia to surround and protect the perforating vessels; **c** Small defect in right rectus abdominis muscle and fascia with a mesh underlay repair (hatched area reflects SeriScaffold® mesh); **d** Areas of rectus abdominis fascial plication

Three months later, the patient was brought to the Operating Room for a laparoscopic left adrenalectomy for the pheochromocytoma. Figure 3 with the patient in position and with complete pharmacologic abdominal relaxation, a Veress needle was placed for insufflation. However, the abdomen proved too tight to accommodate sufficient pneumoperitoneum. Given that inadequate abdominal wall distensibility was responsible for inadequate insufflation, rather than difficulty with Veress needle placement, open trocar placement was not undertaken. A laparoscopic retroperitoneal approach was not attempted because it also relies on creating working space through insufflation-mediated abdominal wall expansion [16–18]. Thus, the laparoscopy was aborted without incision. The adrenalectomy was not converted to an open procedure for several considerations: the patient’s reluctance to proceed with an open intervention; because pharmacological management of pheochromocytoma via alpha blockade rendered the procedure non-emergent [19, 20]; and the belief that primary closure would be troublesome.

While kept on pharmacological management of the pheochromocytoma, the patient was evaluated in the outpatient setting for assessment of abdominal wall compliance at regular intervals. Five months later, the patient’s abdomen was felt to be compliant and she was taken back to the Operating Room for a laparoscopic left adrenalectomy. Pneumoperitoneum was established by Veress needle placement at Palmer’s point and the abdomen accommodated 3 l of pneumoperitoneum. The left adrenalectomy proceeded routinely and the patient was discharged home the following day.

## Discussion

This is the first report in the literature of an aborted laparoscopic procedure for failed pneumoperitoneum following autologous flap-based breast reconstruction. Given the frequency of laparoscopy and abdominal-based breast cancer reconstruction in current surgical practice, this case provides some insight into an important aspect of surgical disease management that has not been recognized or discussed in the literature.

Exposure of the surgical field during laparoscopic surgery is achieved by obtaining peritoneal access in order to create a carbon dioxide pneumoperitoneum. This changes the abdominal wall shape from a cylinder to a dome and expands the abdominal wall surface by 15–20 % [21–23]. The factors that govern the degree of expansion of the surgical working space with pneumoperitoneum continue to be studied, but known factors include age and body size of individuals, as well as the effect of employing neuromuscular blockade, elevated intraabdominal pressures and pre-stretching of the abdominal wall [24].

Difficulties associated with performing laparoscopy in patients who have undergone prior abdominal wall surgery, such as abdominoplasty [1, 2, 4, 25], umbilical hernia repair [26] and transverse rectus abdominis myocutaneous flap-based breast reconstruction [27], along with techniques to overcome such challenges, have been described previously. First, the altered abdominal wall anatomy resulting from previous surgery makes safe and proper trocar placement challenging. The reconstructed umbilicus, which is often the anatomic landmark used to define the midline and inform placement of a Veress needle, may lie left or right of its native location [4]. In such cases, alternate stable landmarks can be used for reference, such as the xiphoid bone or the left subcostal pararectus region, Palmer’s point [28]. In addition, others employ alternate techniques for trocar placement, such as the modified open Hasson technique or the use of direct visual entry systems [25, 27, 29, 30].

Second, trocar perforation can be difficult in patients with previous abdominal surgery if the abdominal wall is fibrotic or scarred [2]. Operative modifications for safe and effective port placement in this setting include providing counter traction with towel clamps and using sharp dissection to perforate fibrosis [4].

Third, prior surgical procedures to tighten the abdominal wall, such as abdominoplasty, can prevent adequate pneumoperitoneum due to diminished abdominal wall compliance. This can result in working space deficiency. Loss of abdominal wall compliance was a major technical limitation described in a study detailing a technical approach to laparoscopic colectomy in patients who had previously undergone abdominoplasty [1]. The loss of abdominal wall compliance was thought secondary to fascial plication and skin removal [1, 4]. To recruit additional abdominal space, some advocate placing patients into the reverse Trendelenburg position with flexion of the legs at the hips [31].

In the present study, unsuccessful laparoscopic transperitoneal adrenalectomy was attempted 3 months following breast reconstruction. The patient’s peritoneal cavity was accessed with a Veress needle but the abdominal wall was not sufficiently compliant to accommodate pneumoperitoneum. It is likely that several factors associated with the patient’s body habitus and the DIEP flap-based breast reconstruction contributed to difficulty establishing pneumoperitoneum. The patient was nulliparous and slender with a 65.9-kg preoperative weight and 21.2 body mass index, and there was minimal fascial relaxation and little excess abdominal soft tissue. Removal of sufficient soft tissue for bilateral breast reconstruction meant that primary closure of the abdominal skin and soft tissue would necessarily be tight. This was exacerbated in this patient with the removal of a segment of rectus fascia and muscle to protect the small caliber perforating vessels to the abdominal wall. On the right side where rectus abdominis muscle and fascia were removed, the defect was repaired with placement of SeriScaffold® mesh beneath the fascia. Studies of synthetic mesh of various composition, including polypropylene, polyester and proline, show that abdominal mesh implantation diminishes abdominal wall compliance [32, 33]. Moreover, the abdominal wall fascia was plicated and therefore tightened at several points: superficial to the mesh underlay to reinforce the repair on the right, in the upper quadrants of the abdomen to reduce postoperatively bulging, and on the left where the small cuff of muscle was excised to protect the microvessels. Fascial plication tightens the abdominal wall, elevates intraabdominal pressures and in extreme cases has been shown to cause abdominal compartment syndrome [34, 35]. Collectively, it is likely that removal of significant abdominal wall tissue in a slender patient, placement of abdominal wall mesh, and fascial plication all contributed to significantly decrease the abdominal wall compliance and preclude sufficient pneumoperitoneum.

Pharmacologic management of pheochromocytoma usually precedes other treatments because it can be immediately life threatening. Alpha-blockade is essential to normalize blood pressure, to expand contracted blood volume and to prevent severe intraoperative hypertension. Beta-blockade may also need to be employed to control heart rate, but only after establishing adequate alpha blockade to avoid unopposed alpha stimulation that can precipitate hypertensive crisis. Once the patient is stabilized medically, operative resection can proceed electively [19, 20, 36]. In this case it was the patient’s preference to undergo breast cancer surgery and reconstruction prior to laparoscopic adrenalectomy, which was safe medically once the patient was fully alpha-blocked. In retrospect, however, delaying the breast surgery until after laparoscopy would have obviated the difficulties associated with abdominal wall compliance.

To date, the appropriate timing of laparoscopic surgery following abdominal wall-based breast reconstruction has not been discussed. In the present case, following the first aborted procedure, the patient was evaluated in the outpatient setting with serial abdominal exams. Over the course of 5 months, the patient’s abdominal wall became notably softer and increasingly pliable. Subsequently, 8 months following breast reconstruction, the patient was taken to the Operating Room for a laparoscopic adrenalectomy where pneumoperitoneum was achieved without difficulty. This case shows that abdominal wall compliance after surgery changes impressively over time and that a return of abdominal wall pliability suitable for pneumoperitoneum is regained.

This report highlights an important area of investigative inquiry. It would be of interest to determine the time course of changes in abdominal wall compliance following abdominal wall surgery. Moreover, it would be important to determine whether objective measures and/or tools exist to assess abdominal wall compliance, similar to what has been described for burn scar assessment and softening [37–39].

For the surgeon scheduling a laparoscopic procedure for a patient who has had prior abdominal wall surgery, including development of flaps for breast reconstruction, preoperative evaluation may indicate that pneumoperitoneum will be problematic. If the laparoscopic procedure is elective, the surgery should be delayed. If not, the informed consent discussion should also include the possibility of aborted procedure for insufficient pneumoperitoneum, or the need for conversion to an open approach.

## Conclusions

Pneumoperitoneum for laparoscopic surgery following autologous deep inferior epigastric (DIEP) perforator flap breast reconstruction may prove difficult as a result of loss of abdominal wall compliance. Surgeons should consider the details of the patient’s breast reconstructive procedure in order to identify patients at risk for difficult pneumoperitoneum. Potential risk factors for difficulty with insufflation after breast reconstruction performed with abdominal tissue include: slender body habitus, nulliparous patient with minimal fascial relaxation, autologous flap-based reconstruction necessitating tight abdominal wall closure, placement of abdominal wall synthetic mesh, and fascial plication to tighten the remaining portions of the abdomen wall.

In the preoperative assessment of patients for laparoscopic surgery following abdominal wall-based breast reconstruction, history and careful abdominal exam to assess abdominal wall stiffness may indicate that laparoscopy for elective procedures should be delayed until abdominal wall compliance normalizes.

### Ethics approval and consent to participate

Not applicable.

### Consent for publication

Written informed consent was obtained from the patient for publication of this report and any accompanying images. A copy of the written consent is available for review by the Editor-in-Chief of this journal.

### Availability of data and materials

Not applicable.

## Abbreviations

DIEP: deep inferior epigastric perforator
