# Erratum to: Failed pneumoperitoneum for laparoscopic surgery following autologous Deep Inferior Epigastric Perforator (DIEP) flap breast reconstruction: a case report

**DOI:** 10.1186/s12893-016-0157-y

**Published:** 2016-07-13

**Authors:** Daniel M. Balkin, Quan-Yang Duh, Gabriel M. Kind, David S. Chang, Mary H McGrath

**Affiliations:** Department of Surgery, Division of Plastic and Reconstructive Surgery, University of California San Francisco, San Francisco, CA USA; Department of Surgery, Section of Endocrine Surgery, University of California San Francisco, San Francisco, CA USA; Department of Plastic Surgery, California-Pacific Medical Center, San Francisco, CA USA

## Erratum

Following publication of the original article in *BMC Surgery* [1], it was brought to our attention that the numbering of Figs. 1 and 3 was switched during the production phase of manuscript publication, and is therefore incorrect.

**Fig. 1:**
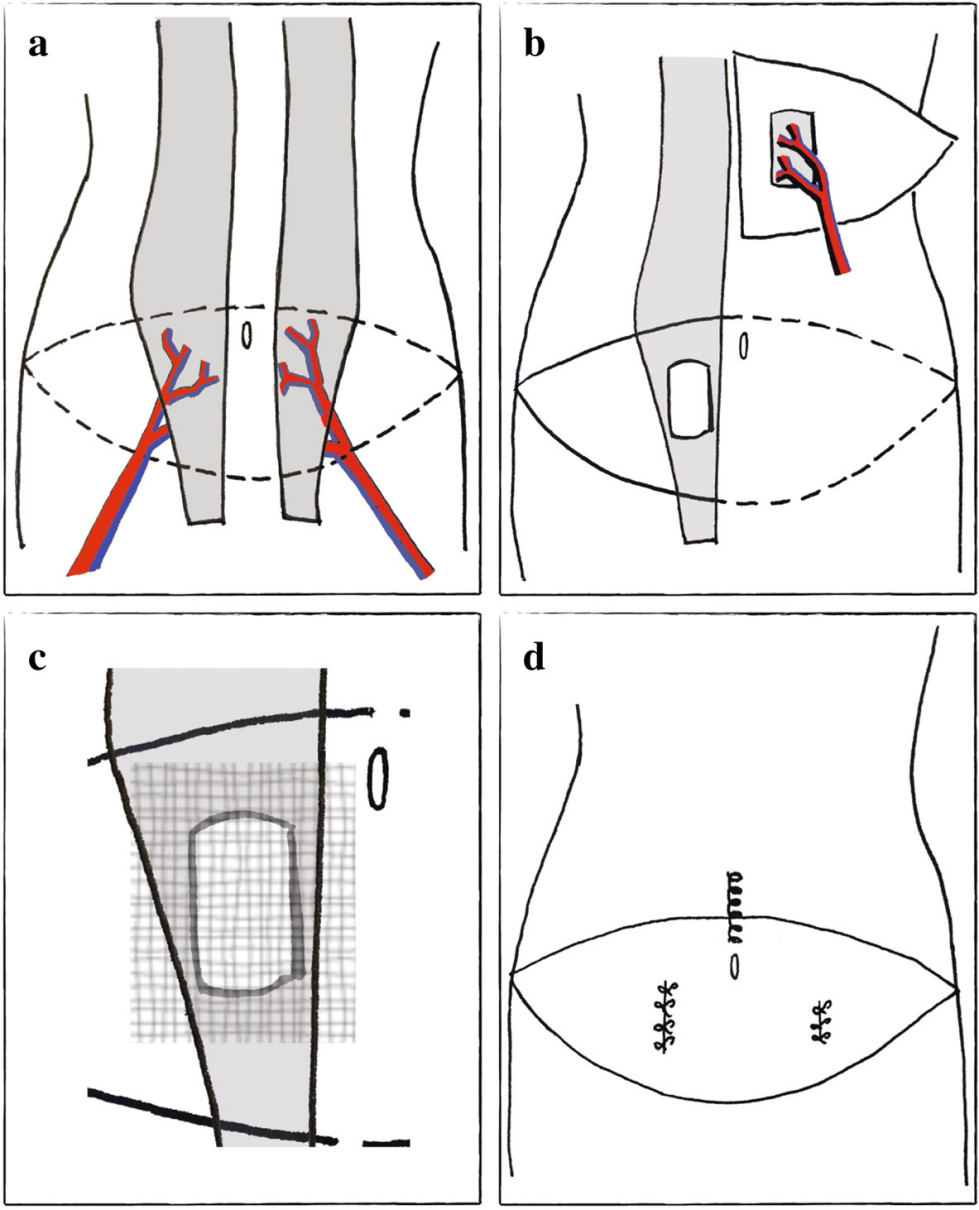
Illustrations of Various Operative Stages of Patient’s Breast Reconstruction. **a** Abdominal wall depicting bilateral rectus abdominis muscles (*grey*) with associated deep inferior epigastric arteries (*red*) and veins (*blue*). Dashed line indicates skin and soft tissue flaps harvested for breast reconstruction; **b** Right-sided DIEP flap used to recreate the left breast mound superimposed over left chest wall. Flap includes a portion of the right rectus abdominis muscle (*grey*) and rectus fascia to surround and protect the perforating vessels; **c** Small defect in right rectus abdominis muscle and fascia with a mesh underlay repair (hatched area reflects SeriScaffold® mesh); **d** Areas of rectus abdominis fascial plication

**Fig. 3:**
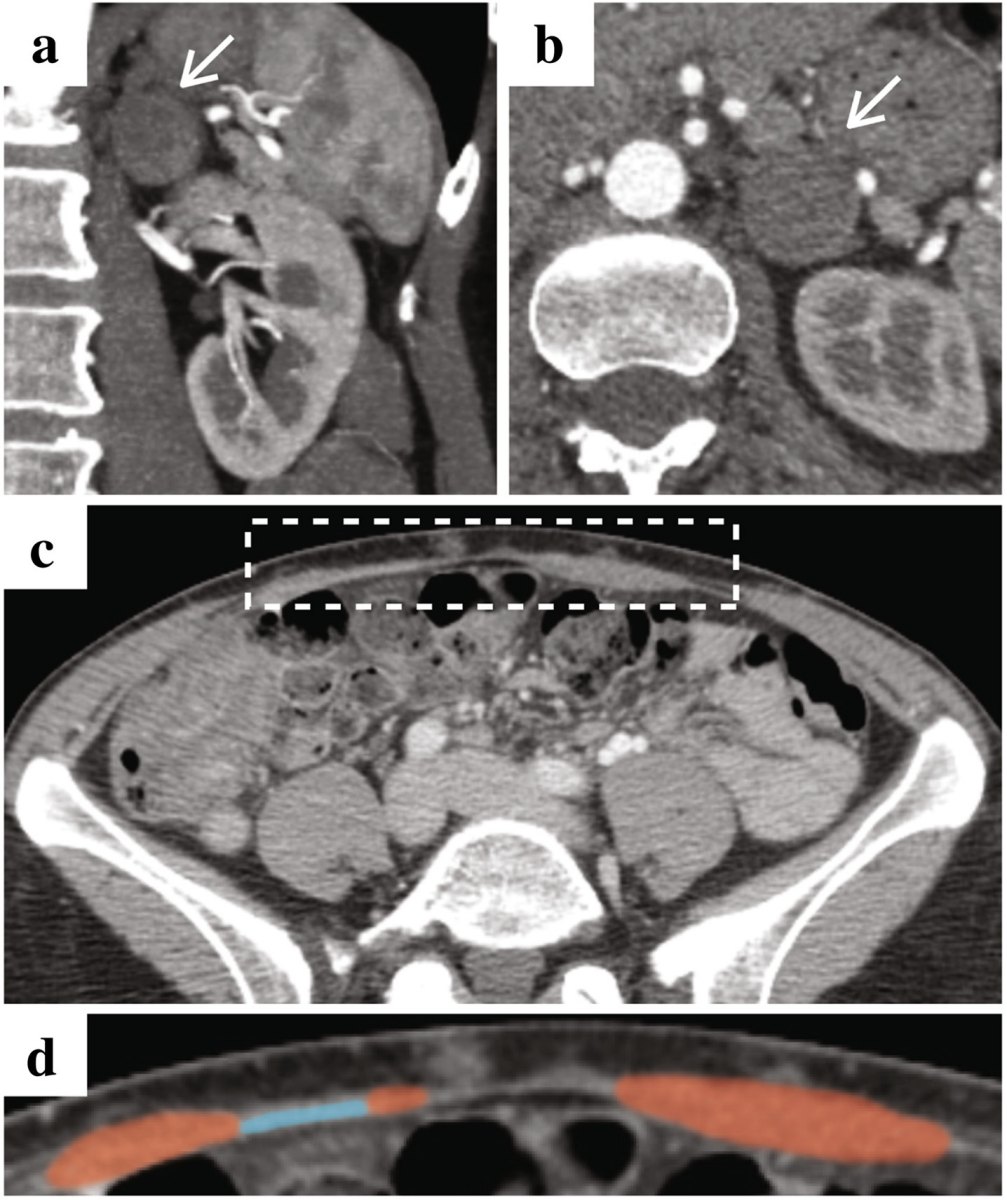
Computed Tomographic Imaging Prior to Laparoscopy Following Breast Surgery. **a** Coronal and **b** axial images demonstration left-sided pheochromocytoma. **c** Low-magnification axial image of the abdomen (*white box* highlights anterior abdominal wall). **d** High-magnification axial image of the anterior abdominal wall (*red* and *blue* indicate rectus abdominis muscle and mesh, respectively)

The correct numbering of the figures should be the following:

- Figure 1. Illustrations of Various Operative Stages of Patient's Breast Reconstruction.
- Figure 2. Patient Photographs Pre and Post Breast Cancer Surgery and Reconstruction.
- Figure 3. Computed Tomographic Imaging Prior to Laparoscopy Following Breast Surgery.

Please find below the figures with the correct numbering. The original article has been updated with the changes. We apologize for the inconvenience this may have caused.
